# Standardizing Quality of Virtual Urgent Care: Using Standardized Patients in a Unique Experiential Onboarding Program

**DOI:** 10.15766/mep_2374-8265.11244

**Published:** 2022-04-12

**Authors:** Daniel J. Sartori, Viraj Lakdawala, Heather B. Levitt, Jason A. Sherwin, Paul A. Testa, Sondra R. Zabar

**Affiliations:** 1 Assistant Professor, Department of Medicine, NYU Grossman School of Medicine; 2 Clinical Associate Professor, Ronald O. Perelman Department of Emergency Medicine, NYU Grossman School of Medicine; 3 Program Manager, Division of General Internal Medicine and Clinical Innovation, Department of Medicine, NYU Langone Health; 4 Associate Director, Virtual Health, Medical Center Information Technology, NYU Langone Health; 5 Assistant Professor, Ronald O. Perelman Department of Emergency Medicine, NYU Grossman School of Medicine; Chief Medical Information Officer, NYU Langone Health; 6 Professor, Division of General Internal Medicine and Clinical Innovation, Department of Medicine, NYU Grossman School of Medicine

**Keywords:** Workplace-Based Assessment, Experiential Onboarding, Emergency Medicine, Primary Care, Simulation, Standardized Patient, Telehealth, Editor's Choice

## Abstract

**Introduction:**

Virtual urgent care (VUC) provides real-time evaluation, triage, and treatment of low-acuity medical problems; however, VUC physicians have varying levels of telemedicine training. We created a workplace-based experiential onboarding program that deployed standardized patients (SPs) into a VUC clinic to evaluate and deliver feedback to independently practicing physicians, providing quality assurance and identifying areas for improvement.

**Methods:**

We simulated evaluation of an adult with upper respiratory symptoms. To replicate a real-life encounter, we developed a mock electronic medical entry with demographic and medical information and scheduled SPs into the clinic's actual patient queue. SPs provided seamless, realistic training within the real-world virtual clinic environment. Using an adapted assessment tool anchored to *not done, partly done,* or *well done,* SPs evaluated communication, disease-specific, and telemedicine skills by observing behaviors. We surveyed participants to evaluate the program.

**Results:**

Twenty-one physicians participated. All performed well in core communication and disease management domains. Ninety-three percent of behaviors (*SD* = 11%) were rated well done within the information gathering domain, 90% (*SD* = 8%) within relationship development, and 95% (*SD* = 5%) within disease management. Physicians struggled with telemedicine-specific skills—55% (*SD* = 38%) well done—and education and counseling—32% (*SD* = 34%) well done—highlighting specific behaviors most ripe for improvement. All queried participants indicated that this simulation improved communication and telemedicine skills.

**Discussion:**

This workplace-based experiential onboarding program uncovered knowledge gaps within telemedicine skills and patient education domains. Identification of these gaps can help drive new virtual care curricula.

## Educational Objectives

By the end of this activity, learners will be able to:

1. Identify gaps in performance of key communication, patient education, and telemedicine-specific skills.
2. Manage a patient presenting with persistent upper respiratory tract symptoms in a virtual care environment according to best practices.
3. Leverage the audio/video interface to augment information gathering and physical examination.
4. Identify individual strengths and weaknesses with utilizing technology in a virtual urgent care visit.

## Introduction

Virtual urgent care (VUC) has become a common means of providing real-time remote evaluation and treatment of low-acuity medical problems. VUC, which leverages audio/video technology to provide care at a distance, can lower barriers to accessibility, promotes appropriate management for common conditions,^1^ and is well liked by patients.^2^ Although virtual care was growing prior to the emergence of COVID-19, the pandemic has solidified the importance of this modality in the care of the chronically and acutely ill, and VUC programs, including our program at NYU Langone Health, have expanded rapidly.^2,3^

However, despite the widespread growth in VUC utilization, there has not been similar expansion in the ways in which VUC providers are trained. VUC programs, ours included, are composed of physicians with heterogeneous clinical backgrounds, most having no formal telemedicine training or experience. Providing virtual care requires unique technical and communication skills distinct from in-person care, including performing virtual physical examinations, querying wearable devices and onsite caregivers, optimizing sound and video, and maintaining appropriate computer etiquette or “webside manner.” Those without prior training in this modality have significant learning gaps.^4^ Some have advocated for adoption of new virtual care core competencies^5^ in an effort to standardize expectations of providers; however, currently few such competencies govern virtual practice.

Therefore, there is the potential for significant variability in the quality of virtual care across providers and across care platforms. Some groups have captured this variability by conducting covert audits of virtual care platforms using secret standardized patients (SPs).^6^ Studies such as these have been critical in unmasking imperfections within the field, with the hope of elevating all VUC physicians to a higher standard of care. However, there still exists an unmet need to provide just-in-time experiential training not only to detect variable quality of virtual care but also to develop and standardize these crucial virtual clinical skills.

Using simulation as a means of teaching and assessing virtual care skills has been done with success previously, within both our institution^4^ and others.^7 9^ Such programs have described OSCEs, which simulate virtual encounters between physicians and SPs. However, while such OSCES provide a useful model for telemedicine training, they are admittedly artificial, deliberately remove the learner from the clinical environment and electronic medical record, impose artificial time constraints, and often use a substitute audio/visual interface. In situ, or workplace-based, simulation, simulating a physician-patient encounter in the live clinical environment, can overcome many of these limitations. By integrating formative low-stakes simulated encounters into physicians' real-world practice setting, workplace-based simulation of virtual care allows for an authentic training experience with the potential to provide more realistic assessments and to make learning virtual care skills more meaningful and practice change more durable. To our knowledge, no such workplace-based virtual care simulation programs have been described.

For several years, our Division of General Internal Medicine and Clinical Innovation has successfully implemented an experiential workplace-based onboarding simulation program to introduce faculty to institutional expectations around communication and patient safety.^10^ While not specific to virtual care, this program utilizes behaviorally anchored checklists, completed by SPs, to evaluate and provide feedback to physicians and other health care providers. The program has been well received by faculty and is a model for skills-based onboarding with the potential to improve patient safety and communication.

Here, we leverage our and others' prior work building simulation-based onboarding and virtual care training programs and adapt it into a workplace-based formative assessment. We describe the development of an experiential onboarding program for VUC physicians in which SPs were deployed into a VUC clinic to evaluate virtual physicians' clinical skills and give individual and program-level feedback, thereby providing quality assurance and identifying areas in need of improvement.

## Methods

### Simulation

We scripted a VUC evaluation of a 36-year-old man with persistent upper respiratory tract symptoms without response to over-the-counter medications (Appendix A). The case encompassed a common presenting concern and potential management dilemma for VUC providers, as virtual visits have been suggested to come with higher rates of antibiotic prescription than in-person encounters.^11^ The case was scripted to challenge the VUC physician to prescribe antibiotics despite symptoms that were inconsistent with a bacterial infection. The case, which simulated a synchronous virtual encounter, was designed to specifically assess virtual care skills. It contained opportunities for physicians to conduct a thorough virtual medicine reconciliation and incorporated prop over-the-counter pill bottles and a prescribed inhaler. The script additionally encouraged a patient-guided virtual physical exam including assessment of vital signs with thermometer, self-check of pulse manually or with wearable device, and visual inspection of the posterior pharynx via phone camera. We hired experienced SPs who, after reviewing a scripted case, underwent 3 hours of dedicated training consisting of rehearsals with authors Daniel J. Sartori and Sondra R. Zabar to familiarize them with the case, props, and anticipated physical exam maneuvers. These authors reviewed each assessment item with the SPs and modeled a range of anticipated behaviors.

In order to faithfully replicate a real-life encounter though this work-place-based assessment, we partnered with our medical center information technology group to create a mock entry in the electronic medical record, which contained the SP's scripted demographic, medical, pharmacy, and allergy information. Each mock entry was associated with a unique username and password. These were used to manually schedule SP appointments with VUC physicians. SP visits were integrated into the VUC physicians' real patient schedule, filling one 15-minute appointment slot (Appendix B). The audio/video interface, which was embedded within the electronic medical record (Epic; Epic Systems), was launched at the start of each visit. SP encounters took place in the authentic virtual clinical environment and in identical fashion to actual patient visits, providing physicians with seamless and realistic training. VUC physician participants, all of whom were residency-trained in emergency medicine, varied in amount of prior clinical experience (between 2 and 23 years), prior virtual experience (0–5 years), and time from hiring to participation in this onboarding simulation (from 1 week to 9 months since hiring). Prior to this project, VUC physicians were given an orientation to the clinic, but there was no standardized virtual care training provided to this population. Encounters took place during VUC physicians' routine 8-hour shifts and were announced to each physician in advance—VUC physicians were notified that appointment slots were designated for SPs to assess their performance. Following each encounter, SP visits were voided such that encounter data were not included in any clinic quality reports.

### Learner Assessment

We had previously developed a behaviorally anchored assessment tool to evaluate core communication skills and telemedicine-specific skills.^4^ Items in this tool were developed from a series of focus groups with experienced telemedicine clinicians, as well as from direct observations of telemedicine encounters. The assessment tool included similar skills described by others, including Cantone and colleagues,^8^ reflecting relevant proposed nursing telehealth Entrustable Professional Activities and skills described by the American Telemedicine Association. Behavioral descriptions were anchored to the following categories: *not done, partly done,* and *well done* (Appendix C). Table details assessment items as well as behavioral descriptors of well-done items. While the encounters were announced, VUC physicians were not primed with the assessment items in advance. SPs offered postencounter verbal feedback to VUC physicians and provided an electronic report of their individual performance as assessed by the checklist described above (Appendix C) within 48 hours of the encounter. VUC program leadership was given a summary of aggregated data of physicians' performance at the project's end. Additionally, all VUC physicians were offered the opportunity to complete a postencounter survey (Appendix D) to provide structured feedback regarding the case. The assessment checklist and survey were entered into the REDCap tool hosted at NYU Langone Health. REDCap,^12,13^ a secure, web-based software platform designed to support data capture for research studies, provided (1) an intuitive interface for validated data capture, (2) audit trails for tracking data manipulation and export procedures, (3) automated export procedures for seamless data downloads to common statistical packages, and (4) procedures for data integration and interoperability with external sources.

**Table.**
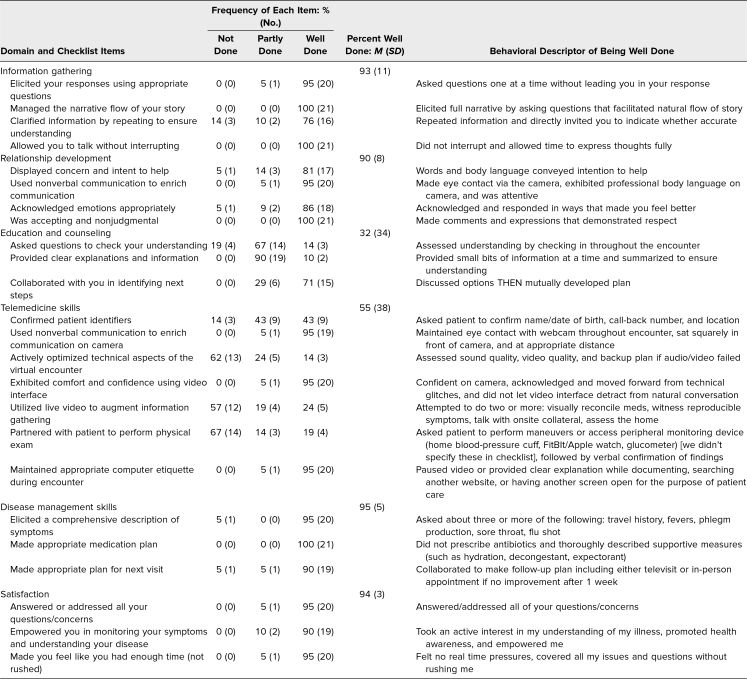
Frequency Distribution of Evaluations for Each Domain and Description of Well-Done Behaviors

## Results

Twenty-one VUC physicians participated in this announced encounter. The physicians performed very well in core communication and disease management domains. Aggregate analysis of SP evaluations demonstrated that 93% of behaviors (*SD* = 11%) were well done within the information gathering domain; 90% (*SD* = 8%) were well done within the relationship development domain (Table). All (100%, *n* = 21) provided appropriate management plans, none of which included antibiotic prescription.

In contrast, education and counseling skills were highly variable and overall less strong, with a mean 32% of behaviors (*SD* = 34%) well done. Within this domain, few (14% of physicians, *n* = 3) received *well done* ratings for checking SPs' understanding, and very few (10%, *n* = 2) provided clear explanations by offering bite-sized pieces of information and summarizing and demonstrating teach-back. Most (71%, *n* = 15) collaborated with the SP to discuss next steps (Table).

Mean telemedicine-specific skills were likewise less strong, with only 55% of behaviors (*SD* = 38%) evaluated as well done. Specific telemedicine skills were used infrequently: Only 19% (*n* = 4) performed an appropriate remote physical exam; 24% (*n* = 5) utilized the audio/video interface to augment information gathering, an item which included virtual medicine reconciliation; and 14% (*n* = 3) optimized technical aspects of the encounter by evaluating sound and/or video or ensuring a backup plan in case the audio/video platform failed (Table).

Satisfaction with the virtual encounter was high. SPs rated 94% of items (*SD* = 3%) as well done within the global satisfaction domain, which included behaviors such as answering SP questions, not rushing the encounter, and empowering health awareness. Twenty out of 21 physicians were rated mostly or completely professional, and the SPs indicated they would recommend or highly recommend based on the physicians' communication skills.

All VUC physicians were given the opportunity to provide structured feedback regarding the case, and a subset of VUC physicians (*n* = 9) did so. All somewhat or strongly agreed that the simulated encounter improved their confidence communicating with patients using the audio/video interface and improved telemedicine skills.

## Discussion

Here, we have described a unique VUC workplace-based assessment utilizing standardized announced encounters to both evaluate and develop the communication and technical skills necessary for virtual care. Given the widespread expansion of virtual care in the wake of the COVID-19 pandemic and the heterogeneity among virtual providers, the need to standardize expectations for virtual care delivery has never been greater. The onboarding program described here provides a realistic and reproducible strategy not only to assess VUC physicians but also to identify specific behaviors in need of improvement and address them with real-time feedback from highly trained SPs. While prior studies employed SPs to assess virtual care quality, few integrated real-time structured feedback into a work-placed based simulation program. To our knowledge, this is the first description of such a program.

VUC physicians excelled in core communication and management domains, including uniformly judicious use of antibiotic regimens, consistent with others' observations of the evolving role of virtual providers as stewards of antibiotic use.^14^ However, we uncovered several areas for improvement, particularly within the domains of telemedicine-specific skills and patient education. Specific telemedicine skills with poorest ratings included using the audio/video interface to augment information gathering and partnering with patients to perform virtual physical examination. Surprisingly, VUC physicians in this project, all hired to provide VUC in a dedicated VUC clinic, struggled with the very skills required to leverage the virtual environment to enhance patient care. Of the many virtues of virtual care, opportunities to visually reconcile medications, assess the home environment, involve other participants, and partner with patients to perform a patient-centered physical examination were often lacking. Our findings suggest that other virtual providers, particularly those more novice in this modality, may demonstrate similar learning gaps.

Education and counseling, which constitute a core component of most physician-patient encounters, were notably executed poorly among most VUC physicians. The assessment items in this domain, which require clear, bite-sized delivery of information and frequent check-back, may represent specific obstacles in the virtual environment and warrant further specific emphasis and training.

This onboarding program was successful given our institution's experience with both implementing SP programs and leveraging the electronic health record to realistically portray virtual SPs in the workplace. Specific early challenges included timing of scheduled SP encounters—scheduling encounters for a visit slot toward the middle or end of each VUC physician's queue made timing unpredictable for SPs due to clinic frequently running behind schedule. Scheduling SPs for the first encounter of each clinic session ameliorated this substantially. Additionally, this project took place when VUC clinic volumes were relatively low, which allowed our group to block off clinic slots without preventing appointments from being scheduled by real patients. Busy VUC clinics looking to replicate a similar workplace-based assessment will need to consider this aspect prior to implementing.

While this project assessed only physicians, several other providers, including nurse practitioners, nurses, and medical assistants, offer virtual care, and future studies can be expanded to include these groups. Two SPs were utilized in this project, but we did not measure interrater reliability, potentially introducing variability in how VUC physicians were assessed. Future studies requiring more SPs to assess larger groups of learners will require attention to standardizing assessments across SPs. As we did not introduce an in-person comparator simulation, we cannot directly assess whether education and counseling or other core communication domain performance measured here is specific to the virtual environment. Lastly, each virtual encounter here was announced to each physician, which potentially could have altered the behaviors being assessed. Future studies using unannounced virtual patients will be needed to faithfully assess physicians' real practice.

Even after the accelerated application of telemedicine levels out in the wake of the pandemic, virtual care will continue to be a necessary and accepted method of providing care. New methods of educating and orienting health care professionals are crucial. This program is generalizable, portable, and scalable and forms a scaffolding on which others can build to test and train other specific skills, onboard new VUC providers, and assure quality control within VUC platforms.

## Appendices


Virtual Urgent Care Visit SP Case.docxPersonnel Responsibilities.docxSP Checklist.docxProgram Evaluation.docx
*All appendices are peer reviewed as integral parts of the Original Publication.*
