# Supplementary material for: Standardizing Quality of Virtual Urgent Care: Using Standardized Patients in a Unique Experiential Onboarding Program
Source: MedEdPORTAL. 2022 Apr 12;18:11244. doi: 10.15766/mep_2374-8265.11244 (PMC9001763; doi:10.15766/mep_2374-8265.11244)
Supplement: Supplementary file 1 — Virtual Urgent Care Visit SP Case.docxPersonnel Responsibilities.docxSP Checklist.docxProgram Evaluation.docx [file mep_2374-8265.11244-s001.zip › A. Virtual Urgent Care Visit SP Case.docx]

Appendix A

Date:June 2019

Primary Case Author: Daniel Sartori, MD

Secondary Case Author: Viraj Lakdawala, MD

Standardized Patient Educator: Sondra Zabar, MD and Daniel Sartori, MD

Name of Case: Virtual Urgent Care Visit

Name of educational and or assessment activity: Workplace-based virtual urgent care simulation

Patient Name: Phillip Stuart

Chief Complaint: Cough and runny nose for 4 days

Most likely Diagnosis and Differential with rationale from history and/or physical exam: Viral upper respiratory tract infection supported by characteristic presentation of non purulent cough, rhinorrhea in the setting of a sick contact.

Challenge question: Do you provide prescription for antibiotics?

Domains: Check all that apply

- Professionalism
- Communication and Interpersonal skills
- Medical History
- Physical exam
- Shared Decision Making
- Patient Education
- Clinical Reasoning

Documentation

Handoff

Presentation

- Other: Appropriate avoidance of antibiotics

Type and level of learner: Virtual urgent care faculty

Case Objectives: please list specific objectives for each of the domains you have checked above:

1. Physician will demonstrate professionalism in interaction with patient, including being respectful, compassionate, accountable and sensitive to patient’s needs.

2. Physician will display concern for patient in a non-judgmental manner and acknowledge patient’s emotions/feelings *through the virtual environment*

3. Physician will elicit full story of patient’s illness, ask appropriate questions and utilize the audio/video interface to augment information gathering

4. Physician will partner with patient to conduct physical exam and elicit comprehensive description of symptoms

5. Physician will collaborate with patient to make plan for treating illness and appropriate follow up

6. Physician will check patient’s understanding by checking in throughout encounter

7. Clinical reasoning: Physician will recognize a common low acuity viral upper respiratory tract infection which can be managed conservatively at home.

8. Physician will not provide prescription for antibiotics

| SETTING: outpatient, in patient, ED, home, nursing home, rehab, group etc. | Outpatient, virtual urgent care video visit with patient at home. |
| --- | --- |
| PATIENT PROFILE: Information about the “patient” that helps select an SP and helps the learner get an understanding of them as a person. SP will know more information about the patient than learner will ever ask but allows SP to portray a fully developed patient personality. If none of the items below are particulars for the case please write “all may be used.” | |
| Age range | 35-40 (SP was told he was 36 years old) |
| Religious/spiritual background | all may be used |
| Sex (e.g., male, female, intersex, transwoman, transman) | Male |
| Sexual Orientation (e.g., heterosexual, lesbian, gay, bisexual, pansexual, queer, asexual) | heterosexual |
| Gender expression (e.g., man, woman, gender queer) | man |
| Race/ethnicity: | White |
| Physical description (e.g., BMI, height range) | Lean, BMI 25 |
| Physical limitations | None, but has not even been able to work from home for 2 days because of illness |
| Patient appearance (e.g., disheveled, hospital gown, business casual, casual) | Casual |
| Moulage + location (e.g., none, bruises, scars, body piercing, tattoos) | None |
| Affect (e.g., pleasant, cooperative) | Pleasant and cooperative but frustrated with feeling so badly. He does not think this is covid-19 as he has been vaccinated x3 and has a negative pcr and home rapid antigen test within the last few days. His family has been planning an event, and have all been extremely cautious and have been testing for covid frequently. He really does not want to miss this event, so strongly requests antibiotic prescription. Has had to sleep on couch for past couple of days because coughing is waking up wife. |
| Family group (e.g., who is family, who they live with) | Lives with wife. |
| Education | College educated |
| Level of health literacy | Has moderate level of health literacy. Has been trying to manage symptoms at home with over the counter medications. Previously used antibiotics for similar sickness. Wife is pediatric ICU nurse, so has access to person with high level of health literacy |
| Employment, if any - present and past, noting any current stresses | Work as an accountant. Is working from home but has barely been able to do this for past 2 days because of feeling so bad |
| Home/homeless - type of dwelling, number of stories, owned or rented | Lives in apartment in East Village in Manhattan |
| Financial situation- any current stresses | Fine, no stresses |
| Insurance Status (e.g., un/under/insured, public/private, HMO/PPO) | Private health insurance |
| Habits (i.e., diet, exercise, caffeine, smoking, alcohol, drugs) | Occasional smoking, alcohol 1 drink, 1-2 times/week |
| Activities (i.e., hobbies, sports, clubs, friends) | Goes out with friends 1-2 times/week, attends family events |
| Typical day - what is the usual daily routine | Typically goes to work in an office but has the option to work from home. He has been working from home the past several days. |

| CASE INFORMATION | |
| --- | --- |
| Chief Concern: What the patient will say when greeted by the student. The patient’s primary reason for seeking medical care often stated in his/own words. | Patient is feeling so bad he didn’t want to leave home for doctor’s visit. Is trying virtual urgent care for first time. Has had a terrible cough for the last 4 days and runny nose. Can’t seem to get better. |
| Additional Concerns: Other, if any, concerns the patient has today (i.e., symptoms, requests, expectations, etc.) that will become part of set agenda. | Patient has had negative SARS-CoV-2 home antigen as well as PCR tests within the last 5 days and doesn’t think this is Covid-19. He has a family event coming up soon and wants to make sure is well enough to attend. His family have all tested negative and he is looking forward to spending time with them. Symptoms have gotten so bad patient has to sleep on the couch so coughing doesn’t wake up wife. |
|  | |
| THE PATIENT STORY: The SP will be asked to tell their symptom story and the personal and emotion impact for each of their concerns. You will want to write this is the patient voice. The symptom story should be able to answer this question: “Tell me more about [chief concern/additional concern], starting at the beginning and bringing me up to now.”  The personal context should be able to answer questions concerning the broader personal/psychosocial context of symptoms, especially the patient beliefs/attributions.  The emotional context should be able to ask how are you doing with this, how does this make you feel, how has this affected you emotionally? IMPACT: How has this affected your life? How has this been for your family? | Patient has had a nagging cough for the past four days. His wife was sick with a cold about a week ago, and at first he thought she gave it to him. Both he and she have tested negative for covid-19 by pcr and home antigen test in the last several days. But he has still had a runny nose and has been coughing without much improvement for three whole days. It has gotten so bad that he has had to sleep on the couch so as not wake her and has not been able to go work for two days.    He has tried an over-the-counter cold medicine, but has not noticed any improvement in symptoms. He has also been using his albuterol metered dose inhaler more frequently than in the past – he is using it twice daily while he uses once weekly at baseline. He has his medicines with him and can demonstrate how he is using / exactly what pills he is taking for the camera. He also has a SARS-CoV-2 home antigen test at home and can show the result of that for the camera as well. He thinks he might have something different than his wife, and possibly worse given that he has not gotten any better. Patient remembers having similar symptoms several years ago and his doctor gave him an antibiotic that began with an ‘A’ – he doesn’t remember exactly what it was called but it worked and he would like to have a prescription for it again now. He is especially concerned because he has a family event coming up this week / weekend and wants to be well for it. |
| HISTORY OF PRESENT ILLNESS: Although some of the HPI will be given in the patient’s symptom story, the learners will expand the story during the direct question section. Below describe the detailed history, usually about the chief concern, which the student must develop in order to make a useful assessment of the problem: | |
|  | |
| Onset (when; gradual or sudden) | Came on over past four days, worse over past 2 days |
| Setting (what was going on or where was patient when symptoms first noticed?) | Living with partner (wife) who had similar symptoms about a week ago. |
| Duration (how long) | Four days |
| Time relationships (frequency, constant or intermittent) | Constant but progressive the last two days |
| Location | Runny nose, cough, clear/yellowish sputum production |
| Radiation | Mild pain in chest with deep coughing |
| Quality | Bothersome |
| Amount | Modest rhinorrhea and phlegm |
| Aggravated by what | Coughing |
| Relieved by what | nothing |
| Associated with what | Feeling achy |
| Attitude (what does the patient think is the problem, and how does he/she feel about it) | Patient is tired of being sick, wants antibiotic prescription because thinks will help and wants to be well enough for family event |
| Overall course | Patient’s above symptoms have persisted for almost four days without relief with over the counter medication and has now made a virtual urgent care appointment which he will take from home. |
| REVIEW OF SYSTEMS: Significant positives and negatives | |
| No measured fever | No vomiting or diarrhea |
| No night sweats or chills | Clear/yellowish drainage from nose |
| Clear, yellowish sputum with cough | Pain in chest with coughing |
| Throat hurts in am, better throughout day |  |
| No difficulty breathing |  |
| No blood in sputum | |
| Past medical history |  |
| Medication allergies (Name and reaction) | None |
| Environmental allergies (Name and reaction) | None |
| Illnesses | Mild intermittent asthma. Acute sinusitis (resolved), |
| Vaccinations | Received flu shot this year, and has had mRNA Covid-19 vaccine x3 (vaccination series plus booster) |
| Surgeries | None |
| Accidents/ injuries/ trauma | None |
| Hospitalization | None |
|  | |
| Inclusive sexual and reproductive history | |
| Sexual practices  Sexual partners  Protection: Use of safer sex practices  Use of birth control if appropriate  Risk of intimate partner violence | All may be used |
| Ob/GYN HISTORY  **N/A, SP is male** | Age of onset of menses  Age of menopause  Number of pregnancies  Number of live births  Number of miscarriages  Number of abortions |
| Medications | Prescription/dose/reason - : Albuterol Metered Dose Inhaler, 2 puffs as needed for shortness of breath or wheeze  Over the counter/dose/reason – cold medication for current cold  Herbs/supplements/dose/reason - None  Other: |
| Immunizations | - Tetanus   **X Flu**   - Hepatitis - Pneumovax - HPV - **X Other SARS-CoV-2 mRNA vaccine x3** |
| Tobacco products:  **X Cigarettes – occasionally when under stress**   - Cigar - Pipe - Chew - E-cigarettes | - Never - Past- year started/year quit - Current   - Quantity   - # of years |
| Alcohol  **X** Beer   - Wine - Liquor - Other | - Never - Past- year started/year quit   **X Current**   - - Quantity: 1 drink, 1-2 times/week   - # of years |
| Drugs   - Weed - Cocaine - Heroin - Meth - Other - IV - Inhalants - Other | **X Never**   - Past- year started/year quit - Current   - Quantity - # of years |
| Diet (describe) | All may be used |
| Exercise (describe) | All may be used |
| List any other important social history or information important to this case | No travel outside of city |
| Family history |  |
| Mother, Father, Siblings, Grandparents, and other significant findings. | All “pretty healthy” |
|  |  |
| Physical Exam- List exam maneuvers expected for this case and any abnormal findings that SP will simulate. (tenderness, hyper-hypo reflex, rebound, weakness etc. )   - Check temperature, in front of the camera - Check pulse, do so at the doctor’s instruction. Patient does not wear an apple watch or fitbit if asked. - Open mouth and shine a light into the back of throat - Feel neck for lumps or bumps - Press on forehead or cheek bones - If asked what your phlegm looks like, cough into a tissue, bring it up to the camera, and state that you are not able to bring up any phlegm at the moment | |
| PHYSICAL EXAM FINDINGS |  |
| 1. Written in layman’s terms | Non-toxic appearing but uncomfortable, coughing |
| 1. General appearance- affect, appearance, position of patient at opening (i.e. sitting, laying down, holding abdomen etc.) | Sitting on the couch, using phone for video visit, sitting squarely in front of the camera. |
| 1. Vital signs | Afebrile (has thermometer and demonstrates this on camera for the physician), P: 80 (checks this for the physician). No BP or oxygen saturation checked. |
| 1. Specific findings and affect | Affect remains a bit pressured throughout the encounter, specific findings elicited by guided physical exam as below. |
| 1. Response to certain physical movements | No posterior oral plaques or exudate, no tender cervical lymphadenopathy, no sinus tenderness. |
|  |  |
| DIAGNOSIS AND DIFFERENTIAL |  |
| Diagnosis with support from positive and negative history and PE findings | Upper respiratory tract viral infection, rule out covid-19, pneumonia and other bacterial infection – supported by non purulent rhinorrhea and sputum, afebrile, without alarm symptoms and very real time negative covid-19 testing. |
|  | Bacterial infection, pneumonia, bronchitis |
|  |  |
| MANAGEMENT OR DIAGNOSTIC PLAN |  |
|  | Continue supportive care and avoid unnecessary antibiotics and an unnecessary in-person visit. Collaborated to make follow up plan including either subsequent televisit or in-person visit if no improvement after 1 week. |
| PROFESSIONALISM ISSUES OR CHALLENGES: | - Telemedicine skills   - Confirmed patient identifiers   - Optimized technical aspects of the virtual encounter   - Exhibited comfort and confidence using video interface   - Utilized live video to augment information gathering, for example asked patient to show medications, witness reproducible symptoms   - Maintained appropriate computer etiquette during encounter - Communication skills   - Appropriate and effective information gathering   - Effectively developed relationship   - Assessed patient’s understanding by checking in with SP throughout the encounter   - Collaborated with patient to make a plan for care   - Made patient feel empowered to monitor and manage symptoms and follow plan |
