# Supplementary material for: Standardizing Quality of Virtual Urgent Care: Using Standardized Patients in a Unique Experiential Onboarding Program
Source: MedEdPORTAL. 2022 Apr 12;18:11244. doi: 10.15766/mep_2374-8265.11244 (PMC9001763; doi:10.15766/mep_2374-8265.11244)
Supplement: Supplementary file 1 — Virtual Urgent Care Visit SP Case.docxPersonnel Responsibilities.docxSP Checklist.docxProgram Evaluation.docx [file mep_2374-8265.11244-s001.zip › B. Personnel Responsibilities.docx]

|  | **Task** | **Personnel** | **Description** |
| --- | --- | --- | --- |
|  | Creation of Mock Patient EHR Entry | Medical Center Information Technology (MCIT) | One test patient entry, with identical properties to real patient entries, was created and published in Epic |
|  | ‘Dressing' Mock Patient | Authors | The test entry was populated with patient demographics, medical history, allergies, home medications, and outpatient pharmacy |
|  | Training SP | Authors | The SP underwent 3 hours of training familiarizing with the case and assessment items, using props (home medicines, tissues) rehearsing virtual physical exam expectations, and tenets of effective feedback. |
|  | Scheduling Visit | Project Manager | Test patients were manually scheduled for 30-minute visit slots and appeared on VUC physician queues identical to real patients. Audio/video interface for telemedicine encounters is embedded within Epic and accessed via hyperlink by the SPs. SPs received mock login credentials to enter the visit virtual waiting room. |
|  | Assessment | Standardized Patient, Virtual Urgent Care (VUC) Physicians | Assessment checklists including behaviorally-anchored evaluation items pertaining to core communication, telemedicine-specific skills, and case-specific performance were transcribed into RedCap and completed by standardized patients remotely. Evaluations regarding the simulation itself were completed by VUC physicians. |
|  | Visit Voiding | Medical Center Information Technology (MCIT) | Visits were voided after completion such that visit data were not included in clinic quality reports. |

**Appendix B.** Personnel Responsibilities
