# Supplementary material for: Standardizing Quality of Virtual Urgent Care: Using Standardized Patients in a Unique Experiential Onboarding Program
Source: MedEdPORTAL. 2022 Apr 12;18:11244. doi: 10.15766/mep_2374-8265.11244 (PMC9001763; doi:10.15766/mep_2374-8265.11244)
Supplement: Supplementary file 1 — Virtual Urgent Care Visit SP Case.docxPersonnel Responsibilities.docxSP Checklist.docxProgram Evaluation.docx [file mep_2374-8265.11244-s001.zip › C. SP Checklist.docx]

**COMMUNICATION**

| ***Information Gathering*** | **Not Done** | **Partially Done** | **Well Done** | *Comments* |
| --- | --- | --- | --- | --- |
| **Elicited** your responses using **appropriate questions** | Asked leading questions AND more than one question at a time | Used leading questions OR asked more than one question at a time | Asked questions one at a time without leading you in your response |  |
| Managed the **narrative flow** of your story | Not able to elicit your story because questions not organized logically | Elicited main elements of story, but illogical order of questions disrupted flow | Elicited full story by asking questions that facilitated natural flow of story |  |
| **Clarified information throughout** by repeating to make sure you were understood | Did not clarify (did not repeat back to you the information you provided) | Repeated info you provided but did not give you a chance to indicate accuracy | Repeated info and directly invited you to indicate accuracy |  |
| Allowed you to talk **without interrupting** | Interrupted | Did not interrupt BUT cut responses short, not enough time | Did not interrupt; Allowed to express thoughts fully |  |
| ***Relationship Develop*** | **Not Done** | **Partially Done** | **Well Done** | *Comments* |
| Displayed **concern and intent to help** | Didn’t communicate concern or intention to help | Words or body language conveyed intention to help | Words and body language conveyed intention to help |  |
| **Used non-verbal communication** to enrich communication | Did only one of the following: 1) made eye contact via the camera, 2) exhibited professional body language on camera, 3) was attentive | Did two of the non-verbal communication items | Did three of the non-verbal communication items |  |
| **Acknowledged emotions/feelings** appropriately | DID NOT acknowledge emotions/feelings | Acknowledged emotions/feelings | Acknowledged & responded in ways that made you feel better |  |
| Was **accepting/non-judgmental** | Made judgmental comments OR facial expressions | Did not express judgment but did not demonstrate respect | Made comments and expressions that demonstrated respect |  |
| ***Educ & Counseling*** | **Not Done** | **Partially Done** | **Well Done** | *Comments* |
| Asked questions to see what you understood (checked your understanding) | Did not check for understanding | Asked if had any questions BUT did not check for understanding | Assessed understanding by checking in throughout the encounter |  |
| Provided **clear explanations**/information | Gave confusing or no explanations | Info was somewhat clear BUT still led to some difficulty in understanding | Provided info in small bits of information AND summarized to make sure clear |  |
| **Collaborated** with you to make a plan | Told you next steps/plan (OR no next steps/plan) | Told you next steps THEN asked patient’s views | Discussed options THEN mutually developed plan |  |

| ***Telemedicine Skills*** | **Not Done** | **Partially Done** | **Well Done** | *Comments* |
| --- | --- | --- | --- | --- |
| Confirmed **patient identifiers** | Did not attempt to identify patient or patient’s location | Asked patient to confirm either a) name and/or date of birth b) call back number OR c) location | Asked patient to confirm a) name and/or date of birth b) call back number AND c) location |  |
| Used non-verbal communication to enrich communication on camera | Avoided eye contact via the webcam, slouched, or was angled away or too far from camera | Made occasional eye contact with webcam, sometimes slouched or out of view | Maintained eye contact with webcam throughout encounter, sat squarely in front of camera, and at appropriate distance |  |
| Actively optimized technical aspects of the virtual encounter | Did not assess sound (e.g. volume, clarity, background noise), video (e.g. pixilation or delay), or ‘backup plan’ if technology failed (e.g. phone call) | Assessed two of the following: sound (e.g. volume, clarity, background noise), video (e.g. pixilation or delay), or ‘backup plan’ if technology failed (e.g. phone call) | Assessed three of the following: sound (e.g. volume, clarity, background noise), video (e.g. pixilation or delay), or ‘backup plan’ if technology failed (e.g. phone call) |  |
| Exhibited comfort and confidence using video interface | Was shy/uncomfortable in front of the camera, let technological glitches (if any) distract from the encounter | Mostly comfortable on camera, occasional stumble but interview was not derailed by occasional video delay or glitch | Confident on camera, acknowledged and moved forward from technical glitches, and did not let video interface detract from natural conversation |  |
| Utilized live video to **augment information gathering** | Made no attempt to visually reconcile medications, witness reproducible symptoms, talk with onsite collateral (family/HHA/VNS) | Did one of the following: visually reconcile medications, witness reproducible symptoms, talk with onsite collateral (family/HHA/VNS) | Did two or more of the following: visually reconcile medications, witness reproducible symptoms, talk with onsite collateral (family/HHA/VNS) |  |
| **Partnered** with patient to **perform virtual physical** exam | Made no attempt to perform physical exam | Asked patient to perform basic exam maneuvers or utilize peripheral monitoring devices (thermometer, home BP cuff,Fitbit/Apple Watch, etc.) | Asked patient to perform maneuvers or access peripheral monitoring device followed by verbal confirmation of findings with patient or collateral |  |
| Maintained **appropriate computer etiquette** during encounter | Frequent typing without explanation, appears preoccupied with computer, or distracted | Occasional type with minimal explanation of actions | Paused video or provided clear explanation while documenting, searching another website, or having another screen open for the purpose of patient care |  |

| ***Case-Specific Assessment*** | **Not Done** | **Partially Done** | **Well Done** | *Comments* |
| --- | --- | --- | --- | --- |
| Elicited a **comprehensive description of symptoms** | Did not ask about fevers, sputum production, sore throat, flu shot | Asked about two of the following: fevers, sputum production, sore throat, flu shot | Asked about three or more of the following: fevers, sputum production, sore throat, flu shot |  |
| Made **appropriate medication plan** | Prescribed antibiotics rather than supportive measures | Did not prescribe antibiotics but did not adequately discuss supportive measures (such as hydration, decongestant, expectorant) | Did not prescribe antibiotics and thoroughly described supportive measures (such as hydration, decongestant, expectorant) |  |
| Made **appropriate plan** for next visit | Did not discuss a follow-up visit | Discussed a less desirable follow-up visit (e.g. immediate presentation to the ED or immediate in-person appointment | Collaborated to make follow-up plan including either televisit or in-person appointment if no improvement after 1 week |  |

**PATIENT SATISFACTION**

| ***The Doctor…*** | **Not Done** | **Partially Done** | **Well Done** | Comments |
| --- | --- | --- | --- | --- |
| Answered or addressed all of your questions/concerns | Answered/addressed only a few of the most central questions/concerns | Answered/addressed many of your questions/concerns | Answered/addressed all of your questions/concerns |  |
| Empowered you in monitoring your symptoms and understanding your illness | Did not make any effort to empower you | Mentioned symptoms to watch out for but did not educate or empower you | Took an active interest in your understanding of your disease, promoted health awareness, and empowered you |  |
| Made you feel like you had enough time (not rushed) | Did not have enough time, visit felt rushed | Mostly had enough time (visit was a bit rushed); felt some time pressure | Felt no real time pressures, covered most of issues and questions without pressure |  |

**Based on communication skills, would you recommend this doctor to a family member or friend?**

| **Not Recommend** | **Recommend with Reservations** | **Recommend** | **Highly Recommend** |
| --- | --- | --- | --- |

**Overall, how would you rate this doctor’s professionalism?**

| **Not at All Professional**  ***Most*** *of the following*   - Disrespectful - Not compassionate - Not accountable - Not sensitive/responsive to my needs/situation | **Somewhat Professional**  *A* ***few*** *of the following*   - Disrespectful - Not compassionate - Not accountable - Not sensitive/responsive to my needs/situation | **Professional**  **3 of the following**   - Respectful - Compassionate - Accountable - Sensitive/responsive to my needs/situation | **Very Professional**  **All of the following**   - Respectful - compassionate - Accountable - Sensitive/responsive to my needs/situation |
| --- | --- | --- | --- |

| SPECIFIC (< 1 min) FEEDBACK: |
| --- |
| COMMENTS (additional remarks, factors affecting your score, impressions) |

This checklist was adapted from the following published work, which appears as citation #4 in the accompanying Educational Summary Report:

Sartori DJ, Hayes RW, Horlick M, Adams JG, Zabar SR. The TeleHealth OSCE: Preparing Trainees to Use Telemedicine as a Tool for Transitions of Care. *J Grad Med Educ*. 2020;12(6):764-768. doi:10.4300/JGME-D-20-00039.1
