# Supplementary material for: Standardizing Quality of Virtual Urgent Care: Using Standardized Patients in a Unique Experiential Onboarding Program
Source: MedEdPORTAL. 2022 Apr 12;18:11244. doi: 10.15766/mep_2374-8265.11244 (PMC9001763; doi:10.15766/mep_2374-8265.11244)
Supplement: Supplementary file 1 — Virtual Urgent Care Visit SP Case.docxPersonnel Responsibilities.docxSP Checklist.docxProgram Evaluation.docx [file mep_2374-8265.11244-s001.zip › D. Program Evaluation.docx]

**Virtual Urgent Care Telehealth SP Encounter**

**For Virtual Urgent Care Physician Participants to Complete**

Thank you for participating in the telehealth SP encounter. We would like to get your feedback on the experience.

*Please answer the following questions:*

|  | *How much has the* ***Virtual Urgent Care Telehealth SP Encounter*** *contributed to your ability to do the following?* | | | |
| --- | --- | --- | --- | --- |
|  | ***Has NOT Contributed AT ALL*** | ***Contributed A LITTLE*** | ***Contributed SOMEWHAT*** | ***Contributed a GREAT DEAL*** |
| Optimize technical aspects of the virtual encounter (ex, adjust sound, video, positioning)? | 🖵 | 🖵 | 🖵 | 🖵 |
| Exhibit comfort and confidence using video interface. | 🖵 | 🖵 | 🖵 | 🖵 |
| Use live video to augment information gathering | 🖵 | 🖵 | 🖵 | 🖵 |
| Partner with patient to perform virtual physical exam. | 🖵 | 🖵 | 🖵 | 🖵 |

*Please indicate the degree to which* ***you agree or disagree with each of the following statements*** *about the Virtual Urgent Care Telehealth SP Encounter:*

|  | *Strongly DISAGREE* | *Somewhat DISAGREE* | *Somewhat AGREE* | *Strongly AGREE* | *Comments*  *(What worked? What didn’t? Suggestions?)* |
| --- | --- | --- | --- | --- | --- |
| Was an effective way to reinforce good habits in health care communication | 🖵 | 🖵 | 🖵 | 🖵 |  |
| Helped me feel more confident about my ability to communicate effectively on video | 🖵 | 🖵 | 🖵 | 🖵 |  |
| Made me more aware of specific telehealth skills | 🖵 | 🖵 | 🖵 | 🖵 |  |
| Helped improve my telehealth skills | 🖵 | 🖵 | 🖵 | 🖵 |  |
| Provided effective feedback from the Standardized Patients | 🖵 | 🖵 | 🖵 | 🖵 |  |
| was well designed | 🖵 | 🖵 | 🖵 | 🖵 |  |
| was a good use of my time | 🖵 | 🖵 | 🖵 | 🖵 |  |
| was engaging | 🖵 | 🖵 | 🖵 | 🖵 |  |

*Overall, to what degree would you recommend this program to a colleague?*

| Not Recommend | Recommend with Reservations | Recommend | Highly Recommend |  |  |
| --- | --- | --- | --- | --- | --- |
| 🖵 | 🖵 | 🖵 | 🖵 |  |  |
| *What is one thing you will do differently after participating in this program?* | | | | | |
| *1)* | | | | | |
| *How can we improve upon this learning activity?* | | | | | |
| *1)* | | | | | |
| *2)* | | | | | |
| *3)* | | | | | |

The above program evaluation is author owned and has not been previously published or adapted.
